# Biliary strictures: diagnostic considerations and approach

**DOI:** 10.1093/gastro/gou072

**Published:** 2014-10-28

**Authors:** Ajaypal Singh, Andres Gelrud, Banke Agarwal

**Affiliations:** ^1^Center for Endoscopic Research and Therapeutics, Division of Gastroenterology, University of Chicago Medical Center, Chicago, IL, USA and ^2^ Division of Gastroenterology and Hepatology, Saint Louis University School of Medicine. St. Louis, MO, USA

**Keywords:** biliary stricture, endoscopic ultrasound, endoscopic retrograde cholangiopancreatography, magnetic resonance cholangio-pancreatography, intraductal ultrasound, cholangioscopy

## Abstract

Biliary strictures present a diagnostic challenge, especially when no etiology can be ascertained after laboratory evaluation, abdominal imaging and endoscopic retrograde cholangiopancreatography (ERCP) sampling. These strictures were traditionally classified as indeterminate strictures, although with advances in endoscopic techniques and better understanding of hepato-biliary pathology, more are being correctly diagnosed. The implications of missing a malignancy in patients with biliary strictures—and hence delaying surgery—are grave but a significant number of patients (up to 20%) undergoing surgery for suspected biliary malignancy can have benign pathology. The diagnostic approach to these patients involves detailed history and physical examination and depends on the presence or absence of jaundice, level of obstruction, and presence or absence of a mass lesion. While abdominal imaging helps to find the level of obstruction and provides a ‘road map' for further endoscopic investigations, tissue diagnosis is usually needed to make decisions on management. Initially ERCP was the only modality to investigate these strictures but now, with the development of endoscopic ultrasound with fine needle aspiration and the availability of newer techniques such as intraductal ultrasound, single-operator cholangioscopy and confocal laser endomicroscopy, the diagnostic approach to biliary strictures has changed significantly. In this review, we will focus on the decision-making process for patients with biliary strictures and discuss the key decision points that should dictate further diagnostic investigations at each step.

## Background

Biliary strictures frequently present a challenge in terms of diagnosis, which requires a multidisciplinary approach. Traditionally, biliary strictures have been considered to be indeterminate when a diagnosis cannot be made after basic laboratory work-up, abdominal imaging and endoscopic retrograde cholangiopancreatography (ERCP) with biliary sampling. Alhough up to 30% of biliary strictures can be benign [1], the vast majority are malignant, the two major malignancies being pancreatic adenocarcinoma and cholangiocarcinoma. Final determination of malignancy in biliary strictures can entail major surgery if pre-operative diagnosis of malignancy cannot be made. The surgical literature suggests that approximately 15–24% patients undergoing surgical resection for suspected biliary malignancy have benign etiology, but there are no clinical or radiological features to reliably distinguish benign from malignant biliary strictures [2–6]. A pre-operative determination of malignancy is therefore highly desirable, to help plan appropriate treatment including the need for- and type of surgery. In this review, we will discuss the etiologies and then focus on radiological and endoscopic work-up of patients with biliary strictures, including the role of emerging technologies.

## Important etiologies of biliary strictures

The exact incidence of biliary strictures is not known and estimates are available only for post-cholecystectomy strictures related to bile duct injuries. Although biliary strictures are usually noted on ERCP or magnetic resonance cholangio-pancreatography (MRCP) in patients with obstructive jaundice, they are sometimes observed in non-jaundiced patients with or without liver chemistry abnormalities. All bile duct strictures in patients with obstructive jaundice should be considered malignant unless a benign etiology is definitively identifiable. The significance of biliary strictures without jaundice is less certain and a much lower proportion of these are malignant. The most common causes of benign biliary strictures include iatrogenic (post liver transplant or cholecystectomy), chronic pancreatitis, primary sclerosing cholangitis, autoimmune diseases (pancreatitis or cholangitis), Mirizzi syndrome and ischemic cholangiopathy (Table 1). Malignant bile duct strictures are usually due to pancreatic adenocarcinoma and cholangiocarcinoma and are less commonly caused by metastatic cancer of the pancreas or liver, ampullary tumors growing into the bile duct, gall bladder cancer obstructing the bile duct or malignant periportal lymph nodes. Pancreatic adenocarcinoma is the most important consideration in patients with distal common bile duct (CBD) strictures. It may present with identifiable mass lesion but in our clinical practice, in about half of patients with pancreatic cancer, a mass lesion is not identifiable by computed tomography (CT) scan or magnetic resonance imaging (MRI) [1, 7]. A mass lesion is usually identifiable by endoscopic ultrasound (EUS) examination in these patients, although a cytological diagnosis of malignancy may be elusive in some cases. Cholangiocarcinoma is the major consideration in patients with stricture of the mid- and proximal extra-hepatic part of the bile duct; hence the main focus in patients with indeterminate bile duct strictures is to rule out an underlying cholangiocarcinoma. The clinical approach to the patient with indeterminate stricture must include a thorough history and physical examination. Particular attention must be paid to the ‘warning signs' that suggest malignancy, including weight loss, abdominal or back pain, worsening performance status or history of recent surgeries (particularly in the past year).

**Table 1.** Etiology of biliary strictures

Benign	Iatrogenic (liver transplant, cholecystectomy)
	Primary sclerosing cholangitis
	Chronic pancreatitis
	Autoimmune pancreatitis
	IgG4 related cholangiopathy
	Autoimmune cholangitis
	Mirizzi Syndrome
	Infections (tuberculosis, viral, parasitic, HIV cholangiopathy)
	Ischemia
	Vasculitis
	Trauma
	Radiation therapy
Malignant	Pancreatic cancer
	Cholangiocarcinoma
	Metastatic disease with external compression (lymph nodes)

## Laboratory testing

Conjugated hyperbilirubinemia with cholestatic pattern of liver chemistries should be looked for in patients with biliary strictures [2, 8]. Patients with obstructive jaundice have a higher likelihood of malignancy than those with normal serum bilirubin levels. Abnormal liver tests, including elevated serum alkaline phosphatase levels, were not associated with greater risk of malignancy in patients with isolated dilation of the CBD [7, 9–12] and we believe that, even in patients with biliary strictures, the presence of abnormal liver chemistries does not have the same clinical significance or risk of malignancy as those with obstructive jaundice. Serum CA19-9 and carcinoembryonic antigen (CEA) are commonly used in clinical practice in patients with suspected hepatobiliary malignancy. Serum CA19-9 levels above 37 U/mL have a sensitivity of approximately 74% in patients with biliary malignancy but the specificity for diagnosing malignancy is unacceptably low when this cut-off is used [8, 13]. Serum CA19-9 levels are also elevated in non-malignant conditions including cholestasis, cholangitis, cirrhosis and stomach cancer [9–12]. Elevated serum CEA level, commonly used as a marker for colorectal adenocarcinoma, has also been shown to have a sensitivity of 33–68% and specificity of 79–95%, for cholangiocarcinoma [13]. Other tumor markers that have been studied for diagnosis of cholangiocarcinoma include transthyretin (TTR), interleukin-6 (IL-6), mucin-5AC (MUC5AC) and matrix metalloproteinase-7 (MMP-7) [14–17]. Their utility in clinical decision-making for management of patients with indeterminate biliary strictures is rather limited and they are not recommended for routine clinical use.

## Radiological tests

### Abdominal ultrasound

Trans-abdominal ultrasonography is usually the initial imaging modality used in patients with suspected biliary obstruction. Its advantages include low cost, ready availability, and safety but the diagnostic yield is user-dependent. Ultrasound has very high sensitivity, approaching 100% in detecting intrahepatic biliary dilatation and the level of obstruction, but it has a very low yield for actual detection of strictures or masses [18–21].

### Computed tomography

Computed tomography imaging has a much higher sensitivity for detecting biliary malignancy than trans-abdominal ultrasound (US) (69% *vs.* 47%), especially for hilar lesions [22]. The development of multi-detector helical scanners, used in conjunction with rapid injection of contrast media, has markedly improved the resolution of CT scans, providing additional information about etiology, based on the rate of contrast uptake and clearance by focal lesions. Ductal infiltrating cholangicarcinoma commonly presents as a biliary stricture without a discreet mass. It appears as a hypo-attenuating lesion during the arterial phase, with enhancement during the delayed phase (this vascular filling pattern is characteristic of desmoplastic reaction seen in bile duct tumors) [23, 24]. The overall sensitivity of CT scan for detection of cholangiocarcinoma ranges from 40–63% but recent studies have suggested a sensitivity of up to 100% in detecting hilar malignancies during the arterial phase [25–27]. However, multi-detector CT (MDCT) cannot reliably differentiate malignant from benign strictures. Studies have shown a sensitivity of 75–80% and specificity of 60–80% for predicting the nature of biliary strictures using CT imaging [28, 29]. An additional advantage of CT scanning is that it provides information about local spread, nodal and vascular involvement, as well as distant metastasis [26]. MDCT can help identify vascular infiltration that determines resectability. Retrospective studies have reported sensitivity and specificity of 86% and 97%, respectively, for detecting arterial invasion and 85% and 97%, respectively, for portal vein involvement. MDCT has 53% sensitivity and 95% specificity for pre-operative determination of regional lymph node involvement [30]. Even though it is not diagnostic of malignancy in many patients with biliary strictures, CT scanning can help in planning further diagnostic evaluation and management.

### Magnetic resonance imaging/ magnetic resonance cholangiopancreatography

**Figure 1.**
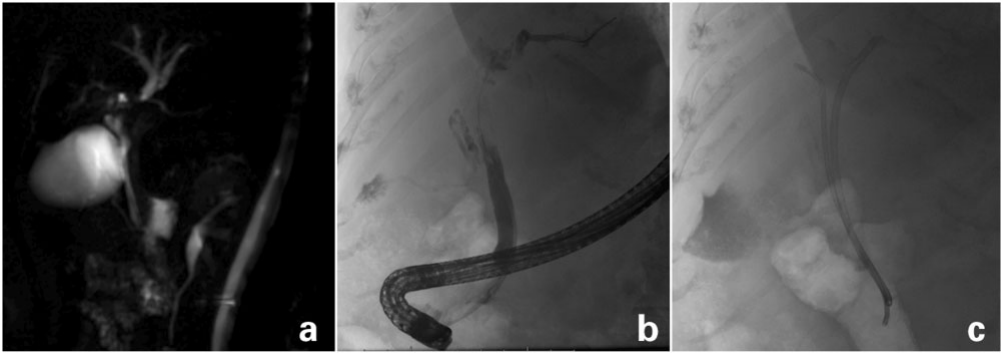
Patient with obstructive jaundice. (a) MRCP showing a hilar stricture and proximal biliary dilatation. (b) ERCP in the same patient before and (c) after placement of two plastic biliary stents.

Magnetic resonance imaging is increasingly being used for cross-sectional imaging of the abdomen in patients with biliary obstruction. The advantages of MRCP over MDCT include the lack of ionizing radiation, along with the ability to provide high quality cholangiograms that can determine the location and extent of biliary strictures and help guide endoscopic therapy, particularly when ERCP is indicated. Due to the risk of acute pancreatitis associated with ERCP, especially in low volume centers, MRCP is preferred for the initial evaluation of patients with biliary strictures. The ability to obtain a cholangiogram without injection of contrast into the biliary tree is significant, since the injection of contrast medium can result in hard-to-treat cholangitis in patients in whom adequate biliary drainage cannot be achieved during ERCP [31, 32]. MRCP has a high reported sensitivity and specificity, similar to that of ERCP, for assessing the level and morphology of biliary strictures [28, 33, 34]. A meta-analysis of 67 studies, including 4711 patients with suspected biliary obstruction, found a sensitivity and specificity of 98% for MRCP in determining the level of obstruction, while the corresponding numbers were 88% and 95% for diagnosing malignancy [35], which can be further improved with the use of diffusion-weighted imaging (DWI) [36]. MRCP can also help in differentiating malignant from benign strictures, with a reported sensitivity of 38–90% and specificity of 70–85% [29, 37, 38], along with 88–96% accuracy in predicting the extent of involvement of the bile duct by cholangiocarcinoma [34, 39]. The disadvantages of using MR imaging include high cost, longer imaging duration, motion artifact, and inability to obtain cytological samples for diagnosis. In our opinion, the choice between MRI/MRCP and high-resolution CT with contrast is based on the suspected location of the biliary stricture according to abdominal ultrasound imaging and institutional expertise. Patients with suspected hilar obstruction (dilated intrahepatic biliary tree without dilation of extrahepatic bile duct) benefit more from MRI/MRCP which, besides locating the stricture and sometimes identifying the mass, can also map the biliary tree (Figure 1). This information is invaluable at the time of ERCP for biliary drainage and attempts at tissue diagnosis with biliary brushings. In patients with extra-hepatic biliary obstruction (dilation of both the intra- and extra-hepatic biliary tree), CT with pancreatic protocol is likely to provide more meaningful information and is preferred in our clinical practice.

## Role of endoscopy in evaluation of biliary strictures

Surgical resection is the preferred option in patients who have a resectable mass with features of cholangiocarcinoma causing biliary obstruction; however surgical resection is associated with significant morbidity and cost. At the time of diagnosis, the vast majority of patients with cholangiocarcinoma have unresectable tumor and cannot undergo potentially curative R0 resection [40]; between 5% and 24% of patients undergoing surgery for suspicious malignant biliary lesions have benign pathology [3, 4]. Pre-operative determination of malignancy in patients with biliary strictures is extremely helpful in the planning of treatment and avoiding exploratory surgery. In patients presenting with painless jaundice and a mass in the head of the pancreas, serological markers for autoimmune pancreatitis must be obtained. A high level of serum IgG4 is a very helpful diagnostic indicator for this rare autoimmune disorder, which has an excellent response to steroids.

ERCP and EUS are two major endoscopic modalities used in the evaluation of patients with biliary obstruction. ERCP was initially the only endoscopic modality for evaluation of biliary strictures, including determination of their etiology, and was routinely used in patients with biliary obstruction. With the advent of EUS and intraductal ultrasound (IDUS), ERCP is not routinely indicated in all patients with biliary obstruction, particularly for the evaluation of distal common bile duct obstruction. Other emerging technologies that have been used in the determining the etiology of biliary strictures include florescent in-situ hybridization (FISH) and confocal laser endomicroscopy (CLE). The use of newer technologies like measurement of volatile organic compounds in the bile duct fluid is being studied to help differentiate benign versus malignant indeterminate strictures [41].

### Endoscopic retrograde cholangiopancreatography

Until recently, ERCP was the most widely used diagnostic procedure in patients with biliary obstruction. Besides identifying the biliary stricture and determining its location and extent, it can provide tissue samples from the stricture for cytological evaluation (brushing- or endoscopic intraductal biopsies, fine needle aspiration, and cytology from removed stents). The stricture morphology is highly unreliable for determining the etiology of the stricture, although long, irregular strictures with shelf-like morphology are considered to suggest malignant biliary strictures. The presence of strictures in both the CBD and pancreatic duct with proximal dilation (double duct sign) is also highly suggestive of a malignant etiology in jaundiced patients [42]. Even though the specificity of diagnosis of malignancy from biliary sampling is high and approaches 95%, the sensitivity is rather low (23–56% for biliary brushings and 33–65% for fluoroscopic biopsies) [27, 43–48]. Burnett *et al*. reviewed 16 studies that included 1556 patients who underwent ERCP with brushing of biliary strictures, and found an overall sensitivity of 41.6% and a negative predictive value of 58% [49]. It has been suggested that the presence of desmoplastic reaction in biliary malignancies contributes to the low yield of cytology [43]. The diagnostic yield for biliary cytology can be increased to 60–70% by using both brushings and biopsies [45, 46]. Multiple biliary brushings can improve the diagnostic yield but neither brush length nor stricture dilation have been shown to be associated with increased yield [50, 51]. The diagnostic yield varies with location, size and type of stricture, cytology preparation and interpretation, as well as the skill and experience of the endoscopist. FISH and flow cytometry have been studied by different investigators with a view to enhancing the diagnostic yield of ERCP cytology. FISH can detect malignant cells in cytology samples by using fluorescence-based polynucleotide probes complimentary to the DNA sequence of interest in the interphase nuclei [52]. The kits currently used employ probes targeting chromosomes 3, 7 and 17 and a probe for the INK4 locus on chromosome 9. FISH was shown to have a high sensitivity and specificity of 84% and 97%, respectively, compared with 22% and 100% for cytology, in diagnosing biliary malignancy [53]. Flow cytometry can help detect malignant cells in cytology samples based on hyperploidy (increased DNA content); it has a similar sensitivity to routine cytology (42%) but a lower specificity (77% *vs.* 92%) [54]. Lankisch *et al*. showed that bile proteomic analysis can successfully differentiate choledocholithiasis from primary sclerosing cholangitis or cholangiocarcinoma, with an area under curve (AUC) of 0.93 (*P = *0.0001; 95% CI 0.82–0.98) and also PSC from cholangiocarcinoma with an AUC of 0.87 (*P = *0.0001; 95% CI 0.73–0.95) [55]. Lipidomic profiling of bile aspirate during ERCP to measure levels of oxidized phospholipids (oxPLs) has been recently used to differentiate cholangiocarcinomas from other benign biliary strictures but further studies to identify the clinically useful oxPLs and to determine their cut-off values are needed [56]. The fluid aspirate from the bile duct can also be analysed for Kras and p53 mutations, to increase the diagnostic yield for malignancy if truly present [57, 58]. Other studies have also shown the possible value of bile aspirate spectroscopy in diagnosing cholangiocarcinoma and presence of S100A9 protein as marker of PSC severity [59, 60]. Bile aspirate analysis is currently not routinely used in work-up of biliary strictures and more studies are required to validate these interesting ideas before the technique is widely used in clinical decision-making. Other technologies, such as cholangioscopy with intra ductal biopsies, confocal endomicroscopy, and narrow band imaging, can also be used during ERCP, [61–63]. Together, these newer techniques promise a higher diagnostic yield for tissue samples obtained during ERCP and, consequently, fewer patients with indeterminate biliary strictures.

### Endoscopic ultrasound

Endoscopic ultrasound is increasingly being used in the diagnostic evaluation of patients with biliary obstruction. It has become the imaging test of choice in patients with distal biliary obstruction, having high sensitivity and accuracy for malignant etiology. The use of EUS-guided fine needle aspiration (EUS-FNA) for the diagnosis of hilar strictures was first proposed in 2000 in a feasibility study of 10 patients with biliary strictures and negative brush cytology [57]. Multiple studies have reported a sensitivity ranging from 40–90%, with most of these showing a sensitivity of more than 70% [64–70]. It is important to note that the majority of these studies included patients with non-conclusive biliary cytology on ERCP [71].

Even though the presence of previously placed stents can cause some acoustic shadowing and/or interfere with the passage of the FNA needle into the area of interest, studies have not shown any significant decrease in diagnostic yield [64]; however, the presence of a biliary stent for several weeks prior to EUS examination can induce inflammatory changes, with accompanying reactive cellular atypia, which can confound the cytological diagnosis and potentially lower the sensitivity and specificity of EUS-FNA in these patients. There has been significant concern over seeding of the FNA tract by malignant cells, especially in patients with proximal and mid-CBD strictures. There are case reports describing needle tract seeding after percutaneous biopsy of hepatocellular carcinoma and percutaneous biliary decompression [72, 73]. A study by Heimbach *et al*. showed that 83% of patients (five out of six) developed peritoneal carcinomatosis after positive transperitoneal biopsies for cholangiocarcinoma [74]. It is important to note that the study included only 16 patients who underwent transperitoneal biopsy and 13 of these were percutaneous biopsies. There are currently no large series reports of needle tract seeding after EUS-FNA of cholangiocarcinoma but seeding of the tract after EUS-FNA of pancreatic adenocarcinoma has been described [75, 76], with a reported incidence of peritoneal carcinomatosis of around 2.2% [77]. These concerns have led to EUS-FNA being considered as a contraindication for liver transplant for chlangiocarcinoma at some transplant centers [78, 79]. A recently published retrospective study by Chafic *et al*. showed no difference in overall- or progression-free survival in patients who underwent EUS-FNA for cholangiocarcinoma than in those who did not undergo FNA [80]. Given the lack of conclusive data, the risk of needle tract seeding should always be discussed with patients undergoing EUS-FNA for biliary obstruction and FNA of proximal/mid CBD strictures should only be performed after ERCP sampling has failed to yield a definitive cytological diagnosis. If there is evidence of tumor spread beyond the bile duct into the surrounding lymph nodes or liver metastasis it is advisable, to help in the management of these patients, to biopsy these lesions for evidence of malignancy. FNA of the biliary stricture may also be considered if there is evidence of vascular infiltration by the tumor during EUS exam.

EUS can also reliably identify alternative etiologies of biliary stricture that do not require surgery, such as periportal lymph node enlargement with malignant infiltration, including those due to lymphoma, lymphomatous involvement of the pancreas or metastatic lesions to the pancreas, especially from the lung and kidney. Occasionally, impacted stones in the bile duct (both in the proximal and distal bile duct) and stones impacted in the cystic duct (Mirizzi syndrome) are diagnosed by EUS in patients with biliary stricture without an identifiable etiology noted on cross-sectional abdominal imaging and ERCP.

### Intraductal ultrasonography

Intraductal ultrasonography involves the insertion into the bile duct of a high-frequency ultrasound probe guided by a wire. It provides high-resolution images of the ductal wall and periductal tissues [81]. Multiple studies have shown a sensitivity and diagnostic accuracy of around 80% and 90%, respectively, for IDUS while evaluating biliary strictures without an associated mass lesion [82–84]. In terms of differentiating malignant from benign strictures, a combination of ERCP and IDUS improved the diagnostic accuracy over that of either ERCP or MRCP alone (88% *vs.* 76% and 58%, respectively) [85]. Meister *et al*. retrospectively reviewed 397 patients with indeterminate biliary strictures who underwent ERCP with IDUS and found IDUS to have sensitivity, specificity and accuracy of 97.6%, 98% and 92%, respectively [86]. Additionally, IDUS can also help in local T and N staging of tumors with accuracies of 84%, 73%, 71% and 68% for T1, T2, T3 and N1 staging, respectively, but it usually underestimates N staging due to the limited penetration of high-frequency ultrasonic waves. Amongst the various characteristics of benign strictures, a bile duct wall thickness of less than 7 mm and the absence of external compression have a negative predictive value of 100% for excluding malignancy in patients with biliary obstruction without a mass, when seen on cross-sectional imaging [87]. Other features suggestive of malignancy, that are identifiable during IDUS examination, include eccentric wall thickening, disruption of the three-layer wall pattern, hypoechoic mass, invasion of surrounding tissue, the presence of lymph nodes and vascular invasion [88–91]. IDUS is potentially an important adjunct in the evaluation of biliary strictures, especially indeterminate strictures, but it has not been widely used because most ERCP practitioners are not trained in EUS and do not feel comfortable interpreting these images. In patients in whom determination of the etiology of the stricture is not possible despite exhaustive evaluation, a repeat ERCP with IDUS examination, performed by an expert endosonographer trained in both ERCP and IDUS, may help identify patients with low likelihood of malignancy, in whom conservative non-surgical management may be reasonable and separate them from patients with high likelihood of malignancy, in whom surgical exploration may be in order.

### Cholangioscopy

**Figure 2.**
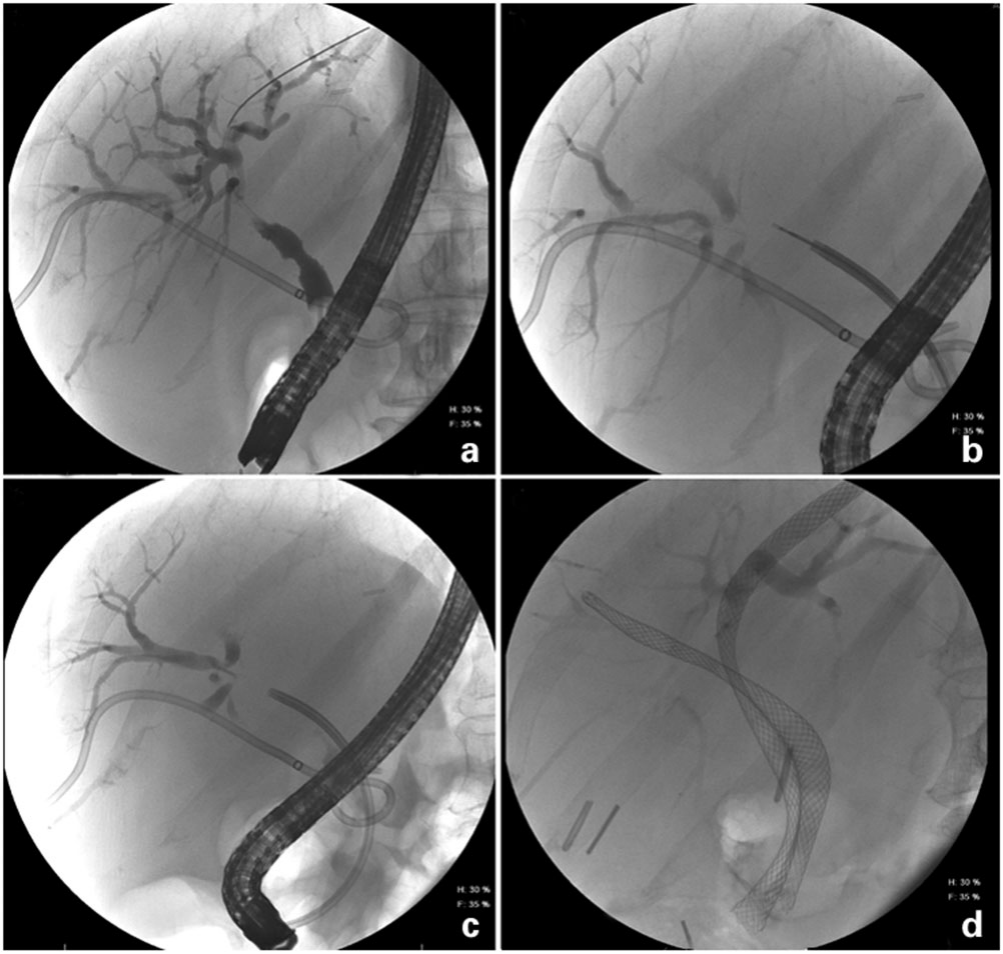
Patient with obstructive jaundice and intrahepatic biliary dilatation noted on abdominal imaging. (a) ERCP showing a biliary stricture with intrahepatic biliary dilatation. (b) Cholangioscopy-guided intraductal biliary biopsy. (c) Fluoroscopic view of cholangioscope. (d) Adequate drainage was obtained after placement of bilateral biliary metal stents.

Direct peroral cholangioscopy was initially introduced in the 1970s but did not gain widespread acceptance, owing to the difficulties associated with use of mother–baby endoscopes: the need for two operators, difficulty with tip maneuverability, time-consuming procedures and fragility of the endoscope [92]. The technique has recently attracted renewed interest from physicians, due to the low sensitivity of brush cytology, the option that it provides of visually guided biopsies, and the development of a single-operator cholangioscopy (SOC) system in 2006 [92–94]. Commonly known as the SpyGlass™ Direct Visualization System or simply SpyGlass (Boston Scientific, Natick, MA, USA), the cholangioscopy catheter can be introduced through the 4.2 mm working channel of a therapeutic duodenoscope after biliary cannulation. It consists of a re-usable 0.77 mm diameter, 6000 pixel optical probe and a disposable 10F access and delivery catheter. The access and delivery catheter has a 0.9 mm channel for the optical probe, a 1.2 mm diameter working channel and two 0.6 mm irrigation channels. Visually directed biopsies can be obtained using a disposable 3 Fr biopsy forceps through the working channel (Figure 2).

In a multicenter feasibility study involving 15 centers in the USA and Europe, SOC was performed in 297 patients, with an overall success rate of 89% and a complication rate of 7.5% over 30 days. But the overall sensitivity and specificity of SOC examination for differentiating malignant and benign ductal abnormalities were 78% and 82%, respectively, higher than the 51% and 54% of ERCP alone; however, the sensitivity of biopsy histology obtained by SOC was only 49% for diagnosis of malignant disease [94]. In another study from Japan, Fukuda *et al*. combined peroral cholangioscopy with ERCP for distinction of malignant and benign strictures and showed a sensitivity of 100%, specificity of 87.5% and an accuracy of 93.5% (positive predictive value = 88.5%; negative predictive value = 100%) [93]; however a standard type of peroral cholangioscope was used in the majority of the cases (84/97) and the catheter-type cholangioscope was used in only 13 of the 97 patients. The advantages of peroral cholangioscopy have been confirmed in other studies as well [93, 95]. Peroral cholangioscopy can also be performed using an ultra-thin endoscope over the wire if a previous sphincterotomy is present. Cholangioscopy is currently not widely used due to the cost and time associated with the procedure, the additional training needed, poor image quality, limited size of working channel and significant risk of cholangitis.

### Confocal laser endomicroscopy

Confocal laser endomicroscopy uses an intravenously injected contrast agent and can provide tissue details at microscopic level in real time, using either a catheter probe that can pass through the working channel of the endoscope (pCLE) or a thin probe advanced through the FNA needle (nCLE). The pCLE catheter can be advanced into the biliary system and can provide visualization of epithelial and subepithelial structures and analyses of capillary blood flow, as well as contrast uptake. The most commonly used contrast agent is fluorescein. The pCLE probe can be passed through various ERCP catheters or the working channel of a cholangioscope. The sensitivity for detecting biliary malignancy ranges from 73–83% but it has low specificity, ranging from 33–50% [63, 96, 97]. A standardized classification system, known as the Miami Classification, was proposed to characterize pCLE findings for biliary strictures. The presence of thick white bands (>20 μm), thick dark bands (>40 μm), dark clumps, epithelial structures and contrast leakage were evaluated as factors that could differentiate malignant from benign strictures [95]. This classification was shown to have a fair-to-poor inter-observer agreement when evaluated by six experienced endoscopists [98]. The low specificity (high false positive numbers) is believed to be secondary to changes associated with chronic inflammation and prior biliary manipulation (stenting, brushing, biopsies); hence a new classification system, called the Paris Classification, was recently described [99]. This includes evaluation of additional features such as vascular congestion, dark glandular patterns, increased interglandular space and thickened reticular structures. More prospective data and validation of a standardized classification system is needed before pCLE can be widely accepted as a useful tool in the diagnostic work-up of biliary strictures.

## A suggested approach to patients with suspected biliary obstruction

**Figure 3.**
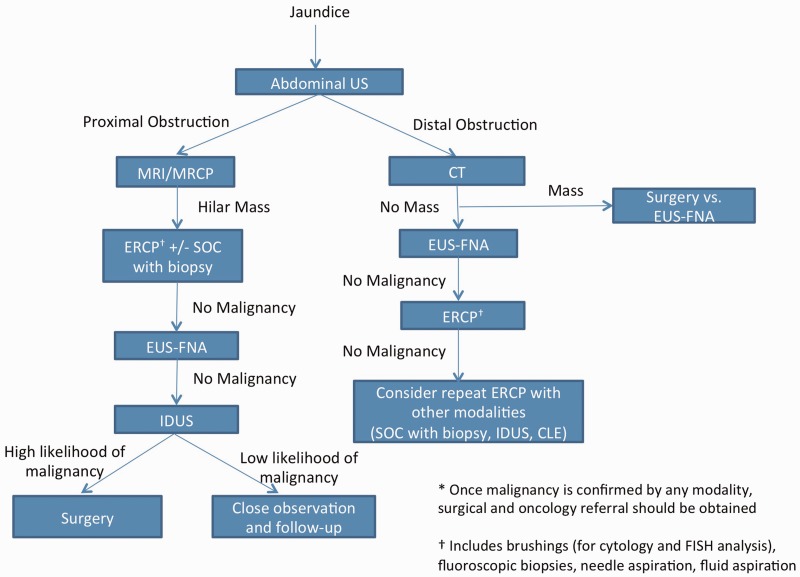
Proposed diagnostic approach to biliary strictures. US = ultrasound; MRI/MRCP = magnetic resonance imaging/magnetic resonance cholangiopancreatography; ERCP = endoscopic retrograde cholangiopancreatography; SOC = single-operator cholangioscopy; EUS-FNA = endoscopic ultrasound with fine needle aspiration; IDUS = intraductal ultrasound; CLE = confocal laser endomicroscopy

With the availability of newer imaging and sampling methods, the algorithms for diagnostic evaluation and management of patients with suspected biliary obstruction have evolved. ERCP is no longer routinely recommended and is not the initial test of choice. Abdominal ultrasound is recommended when looking for gall stones and CBD stones. In the absence of stones, dilation of the intra- and extra-hepatic biliary tree is sought. In patients with only intrahepatic biliary dilation, MRI or MRCP is recommended to search for mass lesions, identify biliary strictures and also look for CBD stones that might have been missed on ultrasound or CT. It can also identify primary sclerosing cholangitis and chronic pancreatitis. In patients with hilar biliary strictures but without an identifiable etiology, MRCP can provide a ‘road map' of the biliary tree, which is helpful during ERCP in these patients. In patients with both intra- and extra-hepatic biliary dilation, CT of the abdomen with pancreatic protocol is usually recommended. In jaundiced patients, if a mass lesion is identified that gives concern over malignancy and CT scan suggests that the mass is resectable, these patients are, in some centers, considered for surgery without attempts at tissue diagnosis and biliary decompression. We earlier reported that, in patients with obstructive jaundice and an identifiable mass lesion on CT scan, 14% are finally diagnosed as free from malignancy. We therefore recommend EUS-FNA in patients with distal biliary obstruction, so as to attempt a tissue diagnosis prior to surgery. The use of EUS-FNA in these patients is predicated on the availability of high-quality EUS and cytological expertise. EUS-FNA in these patients can help identify benign lesions, and also malignant lesions, such as metastatic tumors of the pancreas and primary pancreatic lymphoma, that do not benefit from surgery. In patients with distal biliary obstruction without an identifiable mass lesion, EUS-FNA is recommended as a method of looking for a mass lesion, since up to 40% of malignant neoplasms causing jaundice may be missed on CT scan and can potentially be diagnosed with EUS-FNA. ERCP is recommended in patients with stricture in the proximal and mid-CBD, primarily to obtain tissue for diagnosis. Unless neoadjuvant therapy is planned, biliary decompression by ERCP is not currently recommended in patients with biliary obstruction, due to potentially resectable tumors. ERCP is also indicated in patients with biliary obstruction, in whom the etiology cannot be determined with the aforementioned tests. If malignancy cannot be identified by cytology, FISH, Kras/p53 analysis and flow cytometry should be used (as deemed appropriate by the pathologists), using brushings/biopsies obtained during ERCP. If there is a persistent clinical suspicion for malignancy, a repeat ERCP may be considered in combination with cholangioscopy, to obtain targeted biopsies to improve diagnostic yield. Alternatively, IDUS may be considered in patients with strictures of the proximal and mid-CBD, to look for features that would help differentiate patients with high likelihood of cholangiocarcinoma—who would benefit from surgical exploration—from those with low likelihood of malignancy, in whom conservative, non-operative management may be appropriate (Figure 3).

## Conclusion

In conclusion, biliary strictures remain a diagnostic conundrum and the stakes in achieving an early and accurate diagnosis are high, both due to the risk of failing to spot malignancy and due to the costs and morbidity associated with unnecessary surgery in patients with benign etiologies. A detailed medical history and a multidisciplinary approach, to guide the treatment objectives, is important to assure the best outcome. ERCP with sampling has been the mainstay but it is limited by low sensitivity and the risk of post-ERCP pancreatitis. The addition of FISH, Kras/p53 mutation analysis, intra-ductal biopsies and pCLE may help improve the diagnostic yield but more prospective data are needed. EUS-FNA has been shown to be effective in diagnosing malignancy in patients with biliary strictures, even in the absence of a discrete mass, and should be considered as the initial endoscopic modality in all patients with suspected biliary strictures without obstructive jaundice. Despite limited data, concern remains over needle tract seeding resulting from EUS-FNA, especially if more centers are going to offer transplant as an option for cholangiocarcinoma. Intraductal ultrasound and cholangioscopy may help in patients in whom diagnosis cannot be obtained with above measures, but neither of these modalities is widely used, due to limited experience and availability.

*Conflict of interest statement. * none declared.
